# Assisted Reproductive Technology as a Transcutaneous Route for Bacterial Contamination of Ovarian Endometrioma with Coagulase-Negative Staphylococcus: Case Report and Review of the Literature

**DOI:** 10.1155/2019/4149587

**Published:** 2019-11-29

**Authors:** Shimon Edelstein, Inbar Ben Shachar, Hila Ben-Amram, Seema Biswas, Naama Marcus

**Affiliations:** ^1^Infectious Disease Unit, Ziv Medical Center, Safed, Israel; ^2^Ob/Gyn Department, Ziv Medical Center, Safed, Israel; ^3^The Azrieli Faculty of Medicine, Bar-Ilan University, Safed, Israel; ^4^Clinical Microbiology Lab, Ziv Medical Center, Safed, Israel; ^5^Department of Surgery B, Galilee Medical Center, Nahariya, Israel

## Abstract

Tubo-ovarian abscess may develop in women with endometrioma following assisted reproductive technology (ART). The infection, though rare, is typically late in onset and may present several months after the procedure, and in pregnancy—with the risks of abortion and premature labor. It is thought that transcutaneous oocyte retrieval during ART is the route for bacterial contamination resulting in infection of the endometrioma. Pathogens reported in the literature include *Escherichia coli* (*E. coli*) and Group B streptococcus (GBS) but *Staphylococcus lugdunensis* (*S. lugdunensis*), a coagulase-negative staphylococcus (CoNS), and groin and perineal skin commensal was isolated from the endometrioma in this case. We discuss the challenges in diagnosis and treatment of this rare condition and the implications of the discovery that an organism previously dismissed as a contaminant has emerged as a causative organism in severe, deep-seated infections of soft tissues in recent literature.

## 1. Introduction

The approach to the management of endometrioma before undertaking assisted reproductive technology (ART) has changed over the years. Several decades ago, the management of endometrioma in women undergoing ART included mandatory surgical excision of the endometrioma prior to any ART [1–3]. Several recent reports suggest that preliminary surgery has no effect on ART outcomes and does not increase unresponsiveness to ovarian stimulation [4–6]. Moreover, ovarian reserve may be adversely affected by the surgical excision of endometrioma [7]; thus, current guidelines discourage the excision of endometrioma less than 4 cm in diameter before ART [8, 9]. There is some evidence for nonsurgical ethanol sclerotherapy ablation of endometrioma prior to ART in order to reduce the risk of infection and increase ovarian response [10, 11], the premise, in terms of prevention of infection, being the reduction of volume of substrate for infection. Indeed, in Tsai et al.'s study to estimate the frequency of endometrioma infection following oocyte retrieval, all women underwent concomitant aspiration of the endometrioma (without sclerotherapy) at the time of oocyte retrieval [12].

Because of the risk of importing skin commensals transcutaneously during oocyte retrieval [13], broad-spectrum antibiotic prophylaxis is routinely given at ART. The risk of infection of endometrioma is difficult to quantify, however, as the incidence is so rare. This is reflected in the paucity of literature available on the number of infected endometrioma detected after oocyte retrieval. There are only two studies that have attempted to estimate the frequency of endometrioma infection following oocyte retrieval [12, 14]. Tsai et al. [12] retrospectively reviewed 108 oocyte retrievals performed during a 5-year period and documented only two cases, corresponding to 1.9% (95% CI: 0.3–5.8%). They speculated that pre-surgical vaginal disinfection with povidone iodine might reduce the risk of infection as both cases of infection (pelvic abscess) were observed in the first period of the study—before this measure was implemented (2 out of 56 cases, corresponding to 3.6%, 95% CI: 0.6–10.8%); and no infections were reported after routine vaginal disinfection (none of the 52 cases, corresponding to 0.0%, 95% CI: 0.0–5.5%). This difference, however, did not reach statistical significance.

Benaglia et al. [14] also retrospectively identified women with endometrioma who underwent IVF. These women were contacted by telephone in order to discover the incidence of complications. They reported no incidence of infection after 214 oocyte retrievals, including among women in whom the ovaries were punctured (189 women). The frequency of this complication among the whole cohort was, therefore, 0.0% (95% CI: 0.0–1.7%). Unfortunately, the study was not informative of the risk in the subgroup of women with clinically evident endometrioma puncture as this event was recorded in only six women.

Although rare, the consequences of infection—tubo-ovarian abscess—in women with endometrioma following ART are significant as infection may be discovered several months after the procedure, by which time the women may be pregnant. Outcomes of infection in pregnancy include intrauterine death and premature labor [15–17].

We present a case of infected endometrioma during pregnancy following ART. The rarity and challenge in diagnosis, isolation of the infecting pathogen—usually regarded as a contaminant, and the course of treatment are described with literature review and recommendations.

## 2. Case

A 29-year-old woman, in the 13th week of gestation, was admitted to our gynecological department with right lower abdominal pain. Pregnancy was achieved following an in vitro fertilization prep-implantation genetic diagnostic procedure (IVF/PGD) in a different institution due to known genetic disease. The patient was known to have an endometrioma of 3 cm in the right ovary. This was neither ablated nor excised prior to IVF and IVF/PGD involved the retrieval of 12 oocytes without difficulty. One embryo was transferred.

On admission, 14 weeks after the oocyte retrieval procedure she had diffuse abdominal pain, without fever. White blood count (WBC) was 13 000 per mm^3^, with 84% neutrophils. Transvaginal ultrasound showed a healthy intrauterine pregnancy and right ovarian cyst. The source of infection remained unclear. The following day, the WBC climbed to 17 000 per mm^3^ and she developed local peritoneal signs. Antibiotics (intravenous cefuroxime and metronidazole) were started and an MRI was performed showing a normal appendix and a 5 cm cyst in the right ovary, without free fluid in the pelvis. Despite the normal appearance of the appendix, laparoscopy was performed by the general surgical and gynecological teams. During laparoscopy, pus was seen in the right lower quadrant with adhesion of the tip of an inflamed appendix to the right side of the uterus, which was covered with fibrin and pus. The right ovary was adherent to the pouch of Douglas and difficult to dissect and explore. Suspecting acute appendicitis, an appendectomy was performed. Microbiological samples were taken for culture of the pus, and the intravenous antibiotic prescription continued.

The immediate postoperative course was unremarkable, with a decline in the WBC and some clinical improvement. On the third postoperative day, the abdominal pain recurred and the abdomen became distended. Over the next two days, the WBC began to rise again. Microbiological cultures from the pus aspirated at laparoscopy were negative and the final histopathological report revealed a noninfected appendix, with secondary infection only on the serosal surface.

After reviewing the case, the teams reached the conclusion that the right ovarian cyst (endometrioma) was the probable source of infection. After consultation with the microbiologist, the antibiotic regime was changed to gentamicin, clindamycin, and ampicillin. On the sixth postoperative day, however, the WBC climbed to 30 000 per mm^3^, and a second exploratory laparoscopy was performed by the gynecological team. Upon entry into the peritoneal cavity, greenish free fluid and fibrin were observed in keeping with the clinical picture of generalized peritonitis. Microbiological cultures were taken from the peritoneal fluid. Exploration revealed that the abdominal organs, including the appendix stump, were normal. The right enlarged ovary, however, remained deeply adherent in the pouch of Douglas and was abutting the left ovary. The right ovary was carefully dissected until it was completely freed from the pouch of Douglas. The ovarian capsule was opened, the cyst dissected, and a large volume of pus drained. Microbiological samples of the ovarian pus were sent for culture. Complete cystectomy was precluded by edema of the ovary but fragments of the cyst were submitted for histopathological examination. After drainage, the ovary was left open and the abdomen and pelvis lavaged. A drain was left in the pouch of Douglas.

In the days following the second laparoscopy and drainage of the infected cyst, the patient improved and the WBC decreased to 16 000 per mm^3^. Microbiological cultures from the abdominal fluid were negative, but two bacterial organisms were detected in cultures obtained from the ovarian abscess: *Staphylococcus lugdunensis* (*S. lugdunensis*), and *Peptoccocus anaerobius* (*P. anaerobius*), both of which were sensitive to clindamycin and resistant to penicillin. Antibiotic therapy was, therefore, changed to intravenous clindamycin 900 mg three times daily, gentamicin 240 mg once daily, and cefazolin 1 g three times daily. The patient continued to improve and repeat ultrasound showed a residual ovarian cyst of 3 cm with some heterogeneity within; thus, further surgical intervention as this stage was not considered necessary. The final histopathology report confirmed endometrioid cyst. As *S. lugdunensis* was deemed the causative organism and is associated with severe infection, these antibiotics were continued until discharge after 25 days of hospitalization. On discharge, the patient remained on a 7-day course of oral clindamycin 150 mg and cefalexin 500 mg, both three times daily. She remained under surveillance throughout pregnancy, without further sequelae.

Follow-up ultrasound examination showed a residual endometrioma of 3 cm at 22 weeks gestation with no further change until delivery. The fetus developed normally. A healthy baby of 2975 g was delivered vaginally at term. Four months after delivery, ultrasound demonstrated a substantially smaller cyst in the right ovary measuring 15 × 13 mm.

## 3. Review of the Literature

### 3.1. Infected Endometrioma following ART

A PubMed search for endometrioma/tubo-ovarian abscess (TOA)/infection/ART/IVF/pregnancy revealed only retrospective reports of small case series, a review of 14 cases, and a metanalysis comprising 12 reported cases. In a retrospective analysis by Villette and colleagues [3], ten patients with endometriosis diagnosed at surgery or via standard imaging criteria, with or without endometrioma, underwent emergency hospitalization for tubo-ovarian abscess. Only 3 out of 10 patients, however, had undergone previous oocyte retrieval (16, 57, and 102 days earlier) while the remaining patients appeared to have developed spontaneous endometrioma infection. Treatment included antibiotics and laparoscopic drainage in all patients, with up to 9 days between the initiation of antibiotic therapy and surgery. The isolated bacteria in two patients were *Escherichia coli* (*E. coli*,) and Group B streptococcus (GBS). No bacteria were cultured in the other patient. The authors concluded that (a) infectious complications of ART performed in women with endometriosis may be under-reported because of late onset of the infection, and (b) the best treatment for tubo-ovarian abscess in women with endometriosis following ART is a combination of surgical drainage, as early as possible, and the administration of intravenous antibiotics.

In a meta-analysis performed by Cha Han and colleagues [18], only 38 cases of pelvic abscess were found during pregnancy, only 12 were associated to prior oocyte retrieval. Abortion occurred in 25% of the pregnant women. *E. coli* and Peptostreptococcus species were the most commonly found microorganisms in pelvic abscesses. Eleven of these 12 patients (92%) were treated with a combination of laparotomy/laparoscopic drainage and antibiotics.

Somigliana et al. [13] reviewed the literature for general risks associated with the conservative management of endometrioma less than 4 cm in diameter prior to ART. Among risks of infection, the authors searched for infection of ovarian endometrioma following oocyte retrieval. The development of endometrioma infection following oocyte retrieval in women with ovarian endometrioma was reported by nine independent authors (15–17, 19–23). Overall, 14 cases were described with a range in time from oocyte retrieval to diagnosis of 2–25 weeks. Five patients were pregnant at the time of diagnosis, with poor outcome in 3 of these 5 cases. One patient with twin pregnancy delivered vaginally at 26 weeks. One newborn died [15]. Another patient's pregnancy ended with spontaneous delivery at 22 weeks; the newborn died after birth [16], and another had twin pregnancy delivered by Caesarian Section at 31 weeks [17]. All infections comprised tubo-ovarian abscess and all patients underwent surgical drainage. The authors concluded that with the available evidence, the risk of infection in women with ovarian endometrioma undergoing IVF is very low and does not warrant endometrioma excision prior to ART.

### 3.2. The Pathogen

First described in 1989, by Fleurette et al., [24] *S. lugdunensis* is a part of the normal skin flora, usually found in the groin and perineal regions. Because it lacks coagulase enzymes, it was initially considered a colonizer rather than a real pathogen. It is a Gram-positive bacterium, a CoNS, and a facultative anaerobe. Its morphology is the same as that of other CoNS (like *Staphylococcus epidermidis*), but *in vivo* it acts as if it were coagulase-positive, that is, *Staphylococcus aureus* (*S. aureus*) [25]. Over the last 10 years, *S. lugdunensis* has emerged as a significant pathogen, and several clinical studies have compared its pathogenicity with other coagulase-negative staphylococci causing severe skin and soft-tissue [26] and deep-seated infections [27] such as bone and joint infection, meningitis, catheter-related infection, bacteremia, infective endocarditis, and intra-abdominal infection.

As with *S. aureus*, and unlike other CoNS, *S. lugdunensis* infection can result in high mortality. In contrast to *S. aureus*, however, little is known about the virulence mechanisms of *S. lugdunensis* [28].

*P. anaerobius* was the second bacterium isolated in the case reported here. It is a Gram-positive anaerobic coccus (GPAC), fastidious, and considered to be the most common finding in positive anaerobic bacteremia. There are reports of the involvement of *P. anaerobius* in various sites of infection including the head, respiratory, gastrointestinal and genitourinary tracts, skin and soft tissues, bone and joints, and cardiovascular organs [29–31]. In most of the relevant published articles, infections involving *P. anaerobius* originated mainly from the gastrointestinal and female genitourinary tracts [32–34].

It is likely that the involvement of anaerobic bacteria in infections is underestimated because many of these organisms are slow-growing and fastidious. An added factor is that, in mixed cultures, many anaerobic organisms are easily overgrown by facultative anaerobic bacteria or by any oxygen-consuming bacteria, leading to biased estimates of their true numbers and clinical significance [34]. In most intraperitoneal poly-microbial infections, the anaerobic isolates are considered to be co-pathogens only, and the aerobic bacteria the main pathogen. Thus, while most authorities strongly recommend anti-anaerobic treatment, they still consider the aerobic bacteria to be the real pathogen. In deep-seated, insidious infections of organs sufficiently anatomically or immunologically isolated such that effective concentrations of antibiotic may not be achieved at the site of infection, access of the drug, dosage and duration of antibiotic therapy may be as important for consideration as the pathogens themselves. Accurate microbiological diagnosis in deep-seated infections is, therefore, dependent on retrieval of samples from deep-seated infected tissue, warranting invasive sampling—laparoscopy and drainage of the infected endometrioma in this case.

## 4. Discussion

The key question arising from this case is whether endometrioma infection, rare as it is, should feature prominently in the differential diagnosis of abdominal or pelvic sepsis in a pregnant woman with a history of endometrioma and ART. A second question is whether *S. lugdunensis* should be regarded more commonly as an organism leading to insidious and severe deep-seated pelvic infection, rather than an expected CoNS contaminant of microbiological samples.

### 4.1. Implications for Follow-up after ART

Clearly, there is a need to follow up patients with endometrioma after ART in case of the development of infection. It is not clear how many patients with endometrioma go on to develop infection after transcutaneous oocyte retrieval and how the size of endometrioma affects the risk of sepsis, but, while endometriosis affects around 6–10% of women, the prevalence of endometriosis among women undergoing IVF is as high as 25–35% [35]. Endometrioma is endometriosis in the ovary, but, again, the reports of the prevalence of endometrioma and incidence of endometrioma infection after ART are too few to quantify the odds of infection. Current guidelines recommend conservative management for endometrioma less than 4 cm in diameter prior to ART but these recommendations focus on responsiveness to ovarian stimulation in ART rather than risk of infection after oocyte retrieval. More research is needed on the risks associated with transcutaneous oocyte retrieval, especially where it has been necessary to puncture the endometrioma during the procedure. Also unclear is whether endometrioma infection is spontaneous or as a result contamination during ART.

Although the risk of infection after ART is probably low [13], when it does occur during pregnancy, the outcome may be poor with 25% pregnancies resulting in abortion [18], preterm delivery or infant death [15–17]. As, infection may be diagnosed as late as 25 weeks after oocyte retrieval, in many cases the diagnosis is made after the patient has become pregnant. Thus, it is the consequence of severe infection, especially in pregnancy, rather than the low estimated incidence of infection that informs the clinical follow-up of patients after oocyte retrieval. Women should be informed of the risk of infection, receive broad spectrum antibiotic prophylaxis, and be monitored carefully after the procedure; and in pregnant women, endometrioma infection should feature in the differential diagnosis of acute abdominal pain and sepsis.

### 4.2. S. lugdunensis as a Pathogen

The significance of this first case of endometrioma infection due to *S. lugdunensis* further adds to the debate of whether this CoNS, usually considered a contaminant in microbiological cultures, is a real pathogen as virulent as *S. aureus*. Most CoNS are *S. epidermidis*, the most common sample contaminator. Therefore, one positive culture of CoNS (mostly because only one or two microbiological samples have been received) may mistakenly lead the microbiologist and the clinician to the wrong conclusion of sample contamination as opposed to active infection with CoNS as the causative organism.

### 4.3. Guidelines for the Clinical Management of Endometrioma Infection

What does emerge from this case, and from the few cases in the literature, is that treatment of infection must comprise early surgical drainage. Surgery confirms the diagnosis, permits open drainage of the affected ovary and retrieval of microbiological samples directly from the infected ovarian cyst. Prolonged antibiotic therapy is probably necessary, and therapy should be guided not simply on clinical improvement, but on infected endometrioma culture and sensitivities, and achievement of effective concentrations of the drug at the site of infection. Resistance of *S. lugdunensis* to penicillin and oxacillin has increased in recent years. Our recommendation is combined treatment with clindamycin and cefazolin (or cloxacillin) if the patient presents with mild to moderate sepsis, or with clindamycin and vancomycin (until the final microbiological culture and sensitivity results become available) in severe sepsis.

## 5. Conclusions

This is the first case report to describe *S. lugdunensis* infection of endometrioma during pregnancy following oocyte retrieval. *S. lugdunensis*, previously dismissed as a contaminant in microbiological isolates, has emerged as a pathogen implicated in severe and deep-seated infections. The recommended therapeutic approach in order to mitigate adverse outcomes in pregnancy is early surgical drainage with prolonged antibiotic therapy targeting resistant aerobic and anaerobic bacteria.
